# Complementary and alternative medicine mention and recommendations in inflammatory bowel disease guidelines: systematic review and assessment using AGREE II

**DOI:** 10.1186/s12906-023-04062-0

**Published:** 2023-07-11

**Authors:** Jeremy Y. Ng, Henry Liu, Michelle Chenghuazou Wang

**Affiliations:** Department of Health Research Methods, Evidence, and Impact, Faculty of Health Sciences, McMaster University, Hamilton, ON Canada

**Keywords:** AGREE II, Clinical practice guideline, Complementary and alternative medicine, Crohn’s disease, Inflammatory bowel disease, Ulcerative colitis

## Abstract

**Background:**

Many patients with inflammatory bowel disease (IBD) use complementary and alternative medicine (CAM) for disease management. There is, however, a communication gap between patients and healthcare professionals regarding CAM use, where patients are hesitant to disclose CAM use to providers. The purpose of this study was to identify the quantity and assess the quality of CAM recommendations in IBD clinical practice guidelines (CPGs) using the Appraisal of Guidelines for Research and Evaluation II (AGREE II) instrument.

**Methods:**

MEDLINE, EMBASE, and CINAHL were systematically searched from 2011 to 2022 to find CPGs for the treatment and/or management of IBD. The Guidelines International Network (GIN) and National Center for Complementary and Integrative Health (NCCIH) websites were also searched. Eligible CPGs were assessed using the AGREE II instrument.

**Results:**

Nineteen CPGs made CAM recommendations for IBD and were included in this review. Average scaled domain percentages of CPGs were as follows (overall CPG, CAM section): scope and purpose (91.5%, 91.5%), clarity of presentation (90.3%, 64.0%), editorial independence (57.0%, 57.0%), stakeholder involvement (56.7%, 27.8%), rigour of development (54.7%, 45.9%), and applicability (14.6%, 2.1%).

**Conclusions:**

The majority of CPGs with CAM recommendations were of low quality and their CAM sections scored substantially lower relative to other therapies in the overall CPG. In future updates, CPGs with low scaled-domain percentages could be improved in accordance with AGREE II and other guideline development resources. Further research investigating how CAM therapies can best be incorporated into IBD CPGs is warranted.

**Supplementary Information:**

The online version contains supplementary material available at 10.1186/s12906-023-04062-0.

## Background

Inflammatory bowel disease (IBD) is a family of diseases characterized by chronic inflammation and immunological dysregulation in the gastrointestinal tract which includes but is not limited to Crohn’s disease (CD) and ulcerative colitis (UC) [1]. CD is caused by inflammation of the entire gastrointestinal tract, and it primarily affects the small intestine whereas UC is limited to the large intestine and rectum [2, 3]. Signs and symptoms of both diseases include abdominal pain, diarrhea, fatigue, weight loss, and bloody stools [2]. IBD typically emerges in early adulthood and persists over the patient’s lifespan as continuous cycles of remission and relapse [1, 3]. Due to the chronic nature of this disease, quality of life, social functioning, and the ability to work are all severely impacted for patients with IBD [1, 4]. Additionally, IBD can present a profound psychosocial burden on mental health, with patients describing social isolation, loss of bowel control, impairment of body image, and fear of dependency as factors contributing to emotional distress [5–7]. Patients with IBD have high rates of anxiety and depression [7]; further, one study demonstrated patients with complex IBD may have greater prevalence of depression and poorer perceived health than those with uncomplicated IBD [6]. Approximately 1.5 million Americans and 2.2 million Europeans have been diagnosed with IBD [3, 4], and with increasing prevalence worldwide, IBD is emerging as a new burden on healthcare systems [8]. The etiology of IBD is unknown but complex interactions between genetic susceptibility, age, environment (e.g., stress, diet, or hygiene), and dysbiosis of the gut microbiota all contribute towards the development of IBD [1]. Many current conventional treatments take an anti-inflammatory approach to achieve and maintain remission in IBD, through a wide variety of treatments including immunomodulators, steroids, biologic agents (e.g., monoclonal antibodies), and surgical interventions [9–11]. However, long-term remission and symptom management remains a challenge with many current conventional treatments (e.g., steroids), while often having undesirable adverse effects (e.g., steroid-induced hyperglycemia, increased infection risk) [11, 12]. Correspondingly, patient concerns about treatment adverse effects is one factor associated with treatment noncompliance in many inflammatory conditions (including IBD), which can result in negative patient health outcomes [13]. Further, adverse effects from conventional IBD treatment is a predictor of using complementary and alternative medicine (CAM) among many patients with IBD [14–16].

CAMs are a diverse group of non-mainstream therapies and practices that fall outside the purview of conventional medicine [17, 18]. Complementary medicine refers to non-conventional treatments used in conjunction with conventional treatments, while alternative medicine refers to non-conventional treatments used in place of conventional treatments [17, 18]. While 21 to 60% of patients with IBD have reported CAM use [10, 19–23], many of these patients do not disclose their CAM usage with their healthcare providers [19]. Accordingly, many healthcare professionals have limited knowledge of CAM treatments, where better understanding of the evidence for current IBD CAM treatments can be important for better patient outcomes [10].

Nutritional therapeutics (e.g., herbs and dietary supplements) are the most common CAM therapy used by patients with IBD [10, 19–23]. Among the nutritional therapeutics, probiotics are the most commonly recommended CAM therapy for IBD by gastroenterologists due to its anti-inflammatory and immunomodulatory properties to reduce inflammation of the gastrointestinal tract [10, 19, 23]. One Italian double-blinded randomized control trial demonstrated the efficacy of VSL#3, a probiotic mixture of eight bacterial strains, for IBD [24]. The combination of VSL#3 with conventional medicine (e.g., aminosalicylic acid, immunosuppressants) was more effective in treating IBD than conventional medicine alone [9, 10, 24]. Curcumin, a phytochemical which is commonly found in turmeric, has also been proposed as a CAM therapy for IBD based on its reported anti-inflammatory and anti-oxidative properties on human white blood cells [10, 19, 21–23]. There is, however, limited research on curcumin’s efficacy and dosing [25]. Mind-body practices such as mindfulness, hypnosis, meditation, and yoga are CAM interventions that aim at reducing stress, a potential contributor to IBD development [10, 19–22]. While there is some promising evidence that CAMs may be effective in treating IBD, clinicians generally do not have sufficient training and knowledge to propose or implement CAM regimens to patient treatment plans [23].

Clinical practice guidelines (CPGs) are systematically developed statements often used by healthcare professionals to make recommendations for the treatment and/or management of various conditions, including IBD [26]. Evidence-based CPGs describe guidelines that undertake a systematic literature search, where recommendations are linked to evidence identified through the literature review [27]. Due to insufficient clinician training and expertise in CAMs, CPGs for CAM use in IBD would serve as a beneficial instrument to clinicians working with patients with IBD [28]. To our knowledge, no studies have analyzed the quality of recommendations on CAMs that are found within CPGs for IBD. Therefore, the purpose of this study was to conduct a systematic review to determine the quantity and assess the quality of CPGs providing CAM recommendations made for the treatment and/or management of IBD using the Appraisal of Guidelines for Research and Evaluation II (AGREE II) instrument.

## Methods

### Approach

A systematic review to identify CPGs providing recommendations for the treatment and/or management of IBD was conducted using Cochrane’s standard methods [29] and reported with Preferred Reporting Items for Systematic Reviews and Meta-Analyses (PRISMA) criteria [30]. A protocol for this study was registered with PROSPERO under registration number CRD42020182234. Eligible CPGs with CAM recommendations were assessed twice with the validated AGREE II instrument [31–33], evaluating both the overall CPG and the CPG’s CAM sections. The AGREE II instrument contains 23 items to assess which are grouped into one of the following domains: scope and purpose, stakeholder involvement, rigor of development, clarity of presentation, applicability, and editorial independence.

### Eligibility criteria

Eligible CPGs were those that mention the treatment and/or management of any type of IBD, focusing on populations of adults 18 years of age and older. However, CPGs focusing primarily on special populations (e.g., pediatric, geriatric, pregnant, COVID-19 patients) were excluded. Eligible CPGs were also determined using the following criteria: developed by non-profit organizations (e.g., government agencies, or professional associations or societies); published in 2011 or later, published in English language; and publicly available. CPGs were deemed ineligible if they were published as protocols, abstracts, conference proceedings, letters or editorials; based on primary studies that assessed IBD treatment and/or management; or focused on IBD curriculum, education, training, research, professional certification or performance. If a guideline summary was found, efforts were made to retrieve the full-length guideline, however, the summaries themselves were excluded. Furthermore, if a CPG had been updated multiple times, only the most updated full version of the CPG was assessed. One important note is that the AGREE II instrument was only used to assess eligible CPGs with CAM recommendations in order to establish the difference in scores for CAM-specific sections relative to the entire CPG.

### Searching and screening

MEDLINE, EMBASE, and CINAHL were searched on May 20, 2022, from 2011 to May 19, 2022, inclusive. The search strategy (**Supplementary File** 1) included keywords that reflect terms typically used in the literature to describe IBD. The Guidelines International Network [34], an online repository of guidelines, was searched for eligible CPGs using the following keyword searches: “inflammatory bowel diseases”, “IBD”, “Crohn’s disease”, and “ulcerative colitis.” A search was also performed on the NCCIH website [35], which contains a series of CPGs with CAM recommendations for various conditions. All results were exported into Microsoft Excel for screening. A pilot test for title and abstract screening was performed independently by MCW and HL, followed by an audit by JYN. Following the pilot, MCW and HL screened all titles and abstracts (independently and in duplicate), followed by full text screening by MCW and HL (independently and in duplicate) to evaluate CPG eligibility. Following each step of independent screening, MCW and HL met to resolve discrepancies, and JYN reviewed the screened titles and abstracts and full-text articles, as well as assisted in resolving discrepancies that could not be resolved by MCW and HL.

### Data extraction and analysis

In a data extraction spreadsheet, the following general characteristics were retrieved and summarized from each of the CPGs: publication date; country origin of study; category of organization responsible for publishing the CPG (academic institutions, government agencies, disease-specific foundations, or professional associations or societies); and the presence of CAM mention or recommendations in this guideline (i.e., yes or no). On the condition that CAMs were mentioned in a CPG, the following data were also extracted: category of mentioned CAMs, proposed CAM recommendations, CAM funding sources, and the presence of conflicts of interests (e.g., CAM providers contributing to the guideline panel). To further assess CPG applicability, each developer’s website was evaluated for any knowledge-based resources that corroborated guideline implementation. Data extraction of all CPGs occurred independently and in duplicate by MCW and HL. Following independent data extraction, MCW and HL met to resolve differences; JYN reviewed all extracted data and assisted in resolving any discrepancies unresolved by MCW and HL.

### Guideline quality assessment

Data from eligible CPGs was extracted and analyzed with the AGREE II instrument in accordance with standardized methods [31–33]. JYN, MCW, and HL conducted a pilot test of the AGREE II instrument by independently assessing three CPGs with the AGREE II instrument. All three evaluators met to resolve any discrepancies. Then, all eligible CPGs containing CAM therapy recommendations were assessed twice—once for the overall CPG, and once for the CAM-specific portion of the CPG—by both MCW and HL. All assessments were performed independently and in duplicate. CPGs were scored based on 23 items from six domains using a seven-point Likert scale from strongly disagree (1) to strongly agree (7) to determine if each item was met. Overall quality of the CPG was also rated from 1 to 7, which was used to recommend for or against the use of each CPG. Modified AGREE II questions were piloted by a team of researchers familiar with CPGs prior to the initiative of this study (see **Supplementary File** 2); these modified questions were then used to score the CAM-specific portions of each CPG. JYN helped to resolve scoring discrepancies between MCW and HL. The average assessment scores were determined by computing the average rating of a single evaluator for all 23 items of a single CPG, then averaging this value for both evaluators. The average of both evaluators’ “overall guideline assessment” ratings for each CPG was used to obtain average overall scores. Scaled domain percentages were generated by summing ratings of items within each domain as given by the two evaluators, followed by standardizing the score and converting it to a percentage. Each CPG’s average assessment scores, average overall scores, and scaled domain percentages were compiled for comparison.

## Results

### Search results (Fig. 1)

Searches retrieved 563 items, of which 490 were unique following deduplication. A further 341 items were eliminated based on abstract screening, yielding 149 items whose full texts were considered. Fifty-one items were considered eligible, with 98 items eliminated for the following reasons: 40 were not CPGs, 19 were on a non-IBD topic, 12 were non-English, 16 were CPGs primarily focused on a special population (e.g., pediatric, geriatric, pregnant, COVID-19 patients), 6 were guideline summaries, 4 were not most recent full updated CPGs, and 1 was irretrievable through public access or library systems.

From 51 eligible items [36–86], 26 made no mention of CAM, 4 only made mention of CAM, and 21 made both CAM mention and provided CAM recommendations. One pair of items [37, 38] were considered as one CPG, rather than two, since each item formed the first and second parts of a guideline series. Additionally, a second pair of items [56, 57] were considered as one CPG, as the two articles were dually published but contained identical content. Of these pairs, one pair [37, 38] did not make mention of CAM or provide CAM recommendations, while the other [56, 57] provided CAM recommendations. Hence, in total, there were 49 eligible CPGs, whereby 26 CPGs made no mention of CAM, 4 CPGs only made mention of CAM, and 19 CPGs made both made mention of CAM and provided CAM recommendations.

### Guideline characteristics (Table 1)

**Table 1:** Characteristics of Eligible Guidelines

Guideline	Country	Developer	CAM Category	Guideline Topic
Macaluso et al. 2022 [36]	Italy	Italian Group for the study of Inflammatory Bowel Disease	None	Pharmacologic management of moderate to severe UC
Raine et al. 2022 [37] & Spinelli et al. 2022 [38]	Austria	European Crohn’s and Colitis Organisation	Probiotics	Medical and surgical treatment of UC
De Simone et al. 2021 [39]	Italy; United States	World Society of Emergency Surgery; American Association for the Surgery of Trauma	None	Emergency management of IBD
Feuerstein et al. 2021 [40]	United States	American Gastroenterological Association	None	Pharmacologic management of moderate to severe luminal and perianal fistulizing CD
Holubar et al. 2021 [41]	United States	American Gastroenterological Association	None	Surgical management of UC
Lodyga et al. 2021 [42]	Poland	Polish Society of Gastroenterology; Polish National Consultant in Gastroenterology	Vitamins and Minerals	Management of CD
Miehlke et al. 2021 [43]	Austria; Sweden	United European Gastroenterolog; European Microscopic Colitis Group	Vitamins and Minerals	Management of microscopic colitis
Nakase et al. 2021 [44]	Japan	Japanese Society of Gastroenterology	None	Management of IBD
Adamina et al. 2020 [45]	Austria	European Crohn’s and Colitis Organisation	None	Surgical treatment of CD
Feuerstein et al. 2020 [46]	United States	American Gastroenterological Association	None	Management of moderate to severe UC
Levine et al. 2020 [47]	United States	American Gastroenterological Association	Dietary Supplements	Dietary management of IBD
Colombel et al. 2019 [48]	United States	American Gastroenterological Association	Fecal Microbiota Transplantation, Hypnotherapy, Mindfulness, Probiotics	Management of IBD functional gastrointestinal symptoms
Lightner et al. 2020 [49]	United States	American Society of Colon and Rectal Surgeons	None	Surgical management of CD
Shen et al. 2020 [50]	United States*	Global Interventional Inflammatory Bowel Disease Group	None	Endoscopic treatment of CD
Torres et al. 2020 [51]	Austria	European Crohn’s and Colitis Organisation	None	Pharmacologic treatment of CD
Bonnaud et al. 2019 [52]	France	National Association of the Hepato-gastroenterologists of the National Hospitals Reflexion Club of the Practices and Groups in Hepato-gastroenterology Group for the Study of Treatments for Inflammatory Affectations of the Digestive Tube AFA Crohn-RCH France	None	Management of CD perianal fistulas
Ko et al. 2019 [53]	United States	American Gastroenterological Association	Herbals, Probiotics	Management of mild to moderate UC
Kucharzik et al. 2019 [54]	Germany	German Society for Gastroenterology, Digestive and Metabolic Diseases	Acupuncture, Fecal Microbiota Transplantation, Herbals, Mind-body Medicine, Probiotics	Management of UC
National Institute for Health and Care Excellence (NICE) 2019 [55]	United Kingdom	National Institute for Health and Care Excellence (NICE)	None	Management of UC
Panaccione et al. 2019a [56] & Panaccione et al. 2019b [57]	Canada	Canadian Association of Gastroenterology	Cannabis, Dietary Supplements, Probiotics	Management of luminal CD
Rubin et al. 2019 [58]	United States	American College of Gastroenterology	Herbals, Probiotics	Management of UC in adults
Sood et al. 2019 [59]	India*	Asian Working Group	Dietary Patterns, Probiotics	Dietary management of IBD
Steinhart et al. 2019 [60]	Canada	Canadian Association of Gastroenterology	None	Management of perianal fistulizing CD
Teixeira et al. 2019 [61]	Brazil	Brazilian Medical Association	None	Biologicals treatment of UC
Zaltman et al. 2019 [62]	Brazil	Brazilian Medical Association	None	Biologicals treatment of CD
Bemelman et al. 2018 [63]	Austria	European Crohn’s and Colitis Organisation	None	Surgical treatment of CD
Brown et al. 2018 [64]	United Kingdom	Association of Coloproctology of Great Britain and Ireland	Fecal Microbiota Transplantation, Probiotics	Surgical treatment of IBD
Lichtenstein et al. 2018 [65]	United States	American College of Gastroenterology	Dietary Therapies	Management of CD in adults
Forbes et al. 2017 [66]	Luxembourg	European Society for Clinical Nutrition and Metabolism	Dietary Patterns, Dietary Supplements, Probiotics, Vitamins and Minerals	Nutritional management of IBD
Gionchetti et al. 2017 [67]	Italy	Italian Group for the Study of Inflammatory Bowel Disease	None	Corticosteroid and immunosuppressive treatment of IBD
Nguyen et al. 2017 [68]	United States	American Gastroenterological Association	Probiotics	Management of CD in post-surgical resection patients
Bernstein et al. 2016 [69]	Canada	World Gastroenterology Organisation	Cannabis, Ispaghula, Probiotics	Management of IBD
Harbord et al. 2016 [70]	Austria	European Crohn’s and Colitis Organisation	Vitamins and Minerals	Management of IBD extraintestinal manifestations
Bressler et al. 2015 [71]	Canada	Canadian Association of Gastroenterology	Fecal Microbiota Transplantation, Probiotics	Management of UC in non-hospitalized patients
Eliadou et al. 2015 [72]	New Zealand	New Zealand Society of Gastroenterology	Fecal Microbiota Transplantation	Management of refractory UC
Fichera & Zoccali 2015 [73]	United States	Crohn’s & Colitis Foundation of America	Adipose-derived Stem Cells	Surgical treatment of perianal fistulizing CD
Schwartz et al. 2015 [74]	United States	Crohn’s & Colitis Foundation of America	None	Management of perianal fistulizing CD
Strong et al. 2015 [75]	United States	American Society of Colon and Rectal Surgeons	None	Surgical treatment of CD
Gecse et al. 2014 [76]	United States	World Gastroenterology Organization	None	Management of perianal fistulizing CD
Lee et al. 2014 [77]	United Kingdom	British Dietetic Association	Probiotics	Dietary management of CD
Ross et al. 2014 [78]	United States	American Society of Colon and Rectal Surgeons	Probiotics	Surgical treatment of UC
Gomollon et al. 2013 [79]	Spain	Spanish Group of Ulcerative Colitis and Crohn’s Disease	None	Management of UC
Leung et al. 2013 [80]	China	Hong Kong IBD Society	None	Biological treatment of IBD
Terdiman et al. 2013 [81]	United States	American Gastroenterological Association	None	Thiopurines, methotrexate, and anti-TNF-α treatment of CD
Theede et al. 2013 [82]	Denmark	Danish Society of Gastroenterology and Hepatology	None	Biologicals treatment of IBD
Ueno et al. 2013 [83]	Japan	Guidelines Project Group of the Research Group of Intractable Inflammatory Bowel Disease	Dietary patterns	Management of CD
Bitton et al. 2012 [84]	Canada	Canadian Association of Gastroenterology	None	Treatment of severe UC in hospitalized adults
Mowat et al. 2011 [85]	United Kingdom	British Society of Gastroenterology	Dietary patterns, Probiotics, Vitamins and Minerals	Management of IBD in adults
Orlando et al. 2011 [86]	Italy	Italian Society of Gastroenterology; Italian Group for the study of Inflammatory Bowel Disease	None	Anti-TNF-α treatment of IBD

Eligible CPGs were published from 2011 to 2022 in the United States (n = 17), Austria (n = 5), Canada (n = 5), the United Kingdom (n = 4), Italy (n = 3), Brazil (n = 2), Japan (n = 2), China (n = 1), Denmark (n = 1), France (n = 1), Germany (n = 1), India (n = 1), Luxembourg (n = 1), Poland (n = 1), Spain (n = 1), and New Zealand (n = 1). Additionally, two CPGs had multiple guideline publishing organizations with headquarters based in different countries [39, 43]. Eligible CPGs were funded and/or developed by professional associations or societies (n = 46), disease-specific foundations (n = 2), and a government agency (n = 1). Four CPGs only mentioned CAM, discussing probiotics (n = 3), curcumin (n = 1), dietary therapies (n = 1), and fecal microbiota transplantation (n = 1).

The AGREE II tool was applied to CPGs making CAM recommendations. Of the nineteen CPGs which provided CAM recommendations, these included probiotics (n = 11), fecal microbiota transplantation (n = 5), calcium (n = 4), vitamin D (n = 4), iron (n = 3), cannabis (n = 2), curcumin (n = 2), nutrition therapy (n = 2), omega-3 fatty acids (n = 3), high-fibre diet (n = 2), mind-body medicine (n = 2), acupuncture (n = 1), adipose-derived stem cells (n = 1), chamomile (n = 1), gluten-free diet (n = 1), hypnotherapy (n = 1), ispaghula (n = 1), low-fat diet (n = 1), myrrh (n = 1), other herbal therapies (n = 1), and vegetarian diet (n = 1). Figure 2 summarizes all the CAM recommendations by their corresponding CPGs, for the benefit of clinicians and researchers. Out of the 19 CPGs, only one CPG [45] had CAM practitioners on the guideline development panel.

### Guidelines mentioning CAM without recommendations

There were four CPGs that only made mention of CAM [58, 64, 65, 78]. One CPG noted the short-lasting effects of dietary therapies on CD inflammation reduction [65]. Another discussed a meta-analysis that found no benefits in using probiotics to induce remission, while also briefly mentioning curcumin without further elaboration on efficacy [58]. One CPG mentioned fecal microbiota transplantation as being investigated for treating pouchitis [64]. Three CPGs all discussed the limited evidence showing the probiotic VSL#3 to be effective in maintaining remission for pouchitis in patients with IBD [58, 64, 78].

### Average appraisal scores, average overall assessments and recommendations regarding use of guidelines: overall guideline (Table 2)

**Table 2:** Average Appraisal Scores and Average Overall Assessments of Each Guideline

Guideline	Metric	Appraiser 1	Appraiser 2	Average	Standard Deviation
Raine et al. 2022 [37] & Spinelli et al. 2022 [38] (Overall)	Appraisal Score	5.5	5.5	5.5	0.0
	Overall Assessment	6.0	5.0	5.5	0.7
Raine et al. 2022 [37] & Spinelli et al. 2022 [38] (CAM Section)	Appraisal Score	4.2	4.0	4.1	0.0
	Overall Assessment	4.0	4.0	4.0	0.0
Lodyga et al. 2021 [42] (Overall)	Appraisal Score	3.9	4.0	3.9	0.0
	Overall Assessment	4.0	4.0	4.0	0.0
Lodyga et al. 2021 [42] (CAM Section)	Appraisal Score	3.0	3.3	3.1	0.2
	Overall Assessment	4.0	4.0	4.0	0.0
Miehlke et al. 2021 [43] (Overall)	Appraisal Score	5.0	4.7	4.8	0.2
	Overall Assessment	5.0	5.0	5.0	0.0
Miehlke et al. 2021 [43] (CAM Section)	Appraisal Score	4.1	4.1	4.1	0.0
	Overall Assessment	4.0	4.0	4.0	0.0
Levine et al. 2020 [47] (Overall)	Appraisal Score	3.9	3.8	3.9	0.1
	Overall Assessment	4.0	4.0	4.0	0.0
Levine et al. 2020 [47] (CAM Section)	Appraisal Score	3.3	3.3	3.3	0.0
	Overall Assessment	3.0	3.0	3.0	0.0
Colombel et al. 2019 [48] (Overall)	Appraisal Score	3.7	3.5	3.6	0.2
	Overall Assessment	4.0	3.0	3.5	0.7
Colombel et al. 2019 [48] (CAM Section)	Appraisal Score	3.5	3.1	3.3	0.3
	Overall Assessment	3.0	3.0	3.0	0.0
Ko et al. 2019 [53] (Overall)	Appraisal Score	4.8	4.7	4.8	0.1
	Overall Assessment	5.0	5.0	5.0	0.0
Ko et al. 2019 [53] (CAM Section)	Appraisal Score	4.3	4.0	4.2	0.2
	Overall Assessment	4.0	3.0	3.5	0.7
Kucharzik et al. 2019 [54] (Overall)	Appraisal Score	5.5	5.4	5.5	0.1
	Overall Assessment	6.0	5.0	5.5	0.7
Kucharzik et al. 2019 [54] (CAM Section)	Appraisal Score	4.9	5.0	4.9	0.1
	Overall Assessment	5.0	5.0	5.0	0.0
Panaccione et al. 2019a [56] & Panaccione et al. 2019b [57] (Overall)	Appraisal Score	5.2	5.4	5.3	0.2
	Overall Assessment	5.0	5.0	5.0	0.0
Panaccione et al. 2019a [56] & Panaccione et al. 2019b [57] (CAM Section)	Appraisal Score	4.3	4.6	4.4	0.2
	Overall Assessment	4.0	5.0	4.5	0.7
Sood et al. 2019 [59] (Overall)	Appraisal Score	4.4	4.5	4.4	0.1
	Overall Assessment	4.0	4.0	4.0	0.0
Sood et al. 2019 [59] (CAM Section)	Appraisal Score	3.8	4.0	3.9	0.1
	Overall Assessment	4.0	4.0	4.0	0.0
Forbes et al. 2017 [66] (Overall)	Appraisal Score	4.7	4.7	4.7	0.0
	Overall Assessment	5.0	4.0	4.5	0.7
Forbes et al. 2017 [66] (CAM Section)	Appraisal Score	4.5	4.3	4.4	0.1
	Overall Assessment	4.0	4.0	4.0	0.0
Nguyen et al. 2017 [68] (Overall)	Appraisal Score	4.9	5.0	4.9	0.0
	Overall Assessment	5.0	5.0	5.0	0.0
Nguyen et al. 2017 [68] (CAM Section)	Appraisal Score	4.0	3.8	3.9	0.2
	Overall Assessment	4.0	3.0	3.5	0.7
Bernstein et al. 2016 [69] (Overall)	Appraisal Score	3.5	4.0	3.7	0.3
	Overall Assessment	3.0	4.0	3.5	0.7
Bernstein et al. 2016 [69] (CAM Section)	Appraisal Score	2.9	2.7	2.8	0.2
	Overall Assessment	3.0	3.0	3.0	0.0
Harbord et al. 2016 [70] (Overall)	Appraisal Score	3.8	3.2	3.5	0.5
	Overall Assessment	4.0	3.0	3.5	0.7
Harbord et al. 2016 [70] (CAM Section)	Appraisal Score	3.0	2.7	2.8	0.2
	Overall Assessment	4.0	3.0	3.5	0.7
Bressler et al. 2015 [71] (Overall)	Appraisal Score	4.8	5.0	4.9	0.1
	Overall Assessment	5.0	5.0	5.0	0.0
Bressler et al. 2015 [71] (CAM Section)	Appraisal Score	4.1	4.3	4.2	0.2
	Overall Assessment	4.0	5.0	4.5	0.7
Eliadou et al. 2015 [72] (Overall)	Appraisal Score	3.4	3.6	3.5	0.1
	Overall Assessment	4.0	3.0	3.5	0.7
Eliadou et al. 2015 [72] (CAM Section)	Appraisal Score	2.8	2.7	2.7	0.1
	Overall Assessment	3.0	3.0	3.0	0.0
Fichera & Zoccali 2015 [73] (Overall)	Appraisal Score	3.3	3.0	3.2	0.2
	Overall Assessment	3.0	3.0	3.0	0.0
Fichera & Zoccali 2015 [73] (CAM Section)	Appraisal Score	2.6	2.3	2.5	0.2
	Overall Assessment	3.0	2.0	2.5	0.7
Lee et al. 2014 [77] (Overall)	Appraisal Score	5.0	5.2	5.1	0.1
	Overall Assessment	5.0	5.0	5.0	0.0
Lee et al. 2014 [77] (CAM Section)	Appraisal Score	4.2	4.2	4.2	0.0
	Overall Assessment	4.0	4.0	4.0	0.0
Ueno et al. 2013 [83] (Overall)	Appraisal Score	4.6	4.7	4.6	0.0
	Overall Assessment	4.0	5.0	4.5	0.7
Ueno et al. 2013 [83] (CAM Section)	Appraisal Score	3.7	3.7	3.7	0.0
	Overall Assessment	4.0	4.0	4.0	0.0
Mowat et al. 2011 [85] (Overall)	Appraisal Score	4.7	4.8	4.8	0.1
	Overall Assessment	5.0	5.0	5.0	0.0
Mowat et al. 2011 [85] (CAM Section)	Appraisal Score	3.8	4.0	3.9	0.1
	Overall Assessment	4.0	4.0	4.0	0.0

Average appraisal scores and average overall assessments, evaluated on a seven-point Likert scale, are given in Table 2 for the 19 CPGs assessed using the AGREE II instrument. On the Likert scale, 1 indicates strongly disagreeing, while 7 indicates strongly agreeing, that an item’s criteria were met. Average appraisal scores ranged from 3.2 to 5.5, where 12 CPGs had an average appraisal score of ≥ 4.0 and four CPGs had an average appraisal score of ≥ 5.0. Average overall assessments ranged from 3.0 to 5.5, where 14 CPGs had an average overall assessment of ≥ 4.0 and nine CPGs had an average overall assessment of ≥ 5.0. Five CPGs [48, 69, 70, 72, 73] had an overall assessment of ≤ 4.0.

### Average appraisal scores, average overall assessments and recommendations regarding use of guidelines: CAM sections (Table 2)

Average appraisal scores and average overall assessments for CPGs’ CAM sections, evaluated on a seven-point Likert scale, are shown in Table 2 for the 19 CPGs assessed using the AGREE II instrument. On the Likert scale, 1 indicates strongly disagreeing, while 7 indicates strongly agreeing, that an item’s criteria were met. CAM average appraisal scores ranged from 2.5 to 4.9, where 15 CPGs had an average appraisal score of ≥ 3.0 and eight CPGs had an average appraisal score of ≥ 4.0. Four CPGs [64, 69, 72, 73] had a CAM average appraisal score of ≤ 3.0. CAM average overall assessments ranged from 2.5 to 5.0, with 11 CPGs having an average overall assessment of ≥ 4.0 and only one CPG [54] having an average overall assessment of ≥ 5.0.

### Overall recommendations: overall guideline (Table 3)

**Table 3:** Overall Recommendations for Use of Appraised Guidelines

	Overall Guideline		CAM Section	
Guideline	Appraiser 1	Appraiser 2	Appraiser 1	Appraiser 2
Raine et al. 2022 [37] & Spinelli et al. 2022 [38]	Yes	Yes with Modifications	No	Yes with Modifications
Lodyga et al. 2021 [42]	No	Yes with Modifications	No	Yes with Modifications
Miehlke 2021 [43]	Yes with Modifications	Yes with Modifications	No	Yes with Modifications
Levine et al. 2020 [47]	No	Yes with Modifications	No	No
Colombel et al. 2019 [48]	No	No	No	No
Ko et al. 2019 [53]	Yes with Modifications	Yes with Modifications	No	No
Kucharzik et al. 2019 [54]	Yes	Yes with Modifications	Yes with Modifications	Yes with Modifications
Panaccione et al. 2019a [56] & Panaccione et al. 2019b [57]	Yes with Modifications	Yes with Modifications	No	Yes with Modifications
Sood et al. 2019 [59]	No	Yes with Modifications	No	Yes with Modifications
Forbes et al. 2017 [66]	Yes with Modifications	Yes with Modifications	No	Yes with Modifications
Nguyen et al. 2017 [68]	Yes with Modifications	Yes with Modifications	No	No
Bernstein et al. 2016 [69]	No	Yes with Modifications	No	No
Harbord et al. 2016 [70]	No	No	No	No
Bressler et al. 2015 [71]	Yes with Modifications	Yes with Modifications	No	Yes with Modifications
Eliadou et al. 2015 [72]	No	No	No	No
Fichera & Zoccali 2015 [73]	No	No	No	No
Lee et al. 2014 [77]	Yes with Modifications	Yes with Modifications	No	Yes with Modifications
Ueno et al. 2013 [83]	No	Yes with Modifications	No	Yes with Modifications
Mowat et al. 2011 [85]	Yes with Modifications	Yes with Modifications	No	Yes with Modifications

From the 19 evaluated CPGs, 10 CPGs were recommended for use by both appraisers. Of these 10 CPGs, both appraisers agreed on a rating of “Yes with Modifications” for eight CPGs [43, 53, 56, 57, 66, 68, 71, 77, 85], while appraisers gave different ratings of “Yes” and “Yes with Modifications” for two CPGs [37, 38, 54]. Additionally, both appraisers agreed on a rating of “No” for four CPGs [48, 70, 73, 74], while the remaining five CPGs had conflicting ratings of “Yes with Modifications” and “No” [42, 47, 59, 69, 83].

### Overall recommendations: CAM sections (Table 3)

From the 19 evaluated CPGs, only one CPG’s CAM section was recommended for use by both appraisers [54], where both appraisers agreed on a rating of “Yes with Modifications”. Both appraisers agreed on a rating of “No” for eight CPGs [53, 56, 57, 68–70, 72, 73], while the remaining 10 CPGs had conflicting ratings of “Yes with Modifications” and “No” [37, 38, 42, 43, 56, 57, 59, 66, 71, 77, 83, 85].

### Scaled domain percentage quality assessment (Table 4)

**Table 4:** Scaled Domain Percentages for Appraisers of Each Guideline

Guideline		Domain score (%)					
		Scope and purpose	Stakeholder involvement	Rigour of development	Clarity of presentation	Applicability	Editorial Independence
Raine et al. 2022 [37] & Spinelli et al. 2022 [38]	Overall Guideline	100.0	69.4	86.5	100.0	25.0	58.3
	CAM Section	100.0	27.8	64.6	55.6	2.1	58.3
Lodyga et al. 2021 [42]	Overall Guideline	100.0	30.6	46.9	100.0	14.6	0.0
	CAM Section	100.0	13.9	32.3	69.4	0.0	0.0
Miehlke et al. 2021 [43]	Overall Guideline	88.9	72.2	68.8	91.7	4.2	70.8
	CAM Section	88.9	33.3	63.5	52.8	2.1	70.8
Levine et al. 2020 [47]	Overall Guideline	91.7	47.2	43.8	77.8	0.0	50.0
	CAM Section	91.7	33.3	29.2	58.3	0.0	50.0
Colombel et al. 2019 [48]	Overall Guideline	86.1	44.4	26.0	94.4	6.25	50.0
	CAM Section	86.1	27.8	21.9	91.7	0.0	50
Ko et al. 2019 [53]	Overall Guideline	94.4	50.0	65.6	94.4	8.3	83.3
	CAM Section	94.4	25.0	59.4	69.4	0.0	83.3
Kucharzik et al. 2019 [54]	Overall Guideline	100.0	86.1	67.7	100.0	35.4	87.5
	CAM Section	100.0	94.4	54.2	94.4	6.3	87.5
Panaccione et al. 2019a [56] & Panaccione et al. 2019b [57]	Overall Guideline	100.0	91.7	67.7	100.0	10.4	91.7
	CAM Section	100.0	33.3	62.5	77.8	0.0	91.7
Sood et al. 2019 [59]	Overall Guideline	91.7	38.9	58.3	83.3	14.6	75.0
	CAM Section	91.7	13.9	52.1	75.0	0.0	75.0
Forbes et al. 2017 [66]	Overall Guideline	86.1	30.6	60.4	94.4	27.1	100.0
	CAM Section	86.1	13.9	60.4	88.9	12.5	100.0
Nguyen et al. 2017 [68]	Overall Guideline	91.7	63.9	68.8	94.4	18.8	66.7
	CAM Section	91.7	22.2	60.4	55.6	0.0	66.7
Bernstein et al. 2016 [69]	Overall Guideline	97.2	52.8	28.1	75.0	25.0	25.0
	CAM Section	97.2	30.6	17.7	33.3	0.0	25.0
Harbord et al. 2016 [70]	Overall Guideline	80.6	33.3	29.2	91.7	12.5	29.2
	CAM Section	80.6	8.3	20.8	61.1	8.3	29.2
Bressleret al. 2015 [71]	Overall Guideline	97.2	58.3	66.7	100.0	4.2	91.7
	CAM Section	97.2	22.2	60.4	72.2	0.0	91.7
Eliadou et al. 2015 [72]	Overall Guideline	100.0	44.4	28.1	69.4	10.4	25.0
	CAM Section	100.0	16.7	22.9	27.8	0.0	25.0
Fichera & Zoccali 2015 [73]	Overall Guideline	72.2	30.6	34.4	86.1	0.0	0.0
	CAM Section	72.2	11.1	25.0	36.1	0.0	0.0
Lee et al. 2014 [77]	Overall Guideline	100.0	80.6	76.0	83.3	18.8	45.8
	CAM Section	100.0	33.3	72.9	47.2	4.2	45.8
Ueno et al. 2013 [83]	Overall Guideline	83.3	80.6	56.3	88.9	4.2	83.3
	CAM Section	83.3	33.3	43.8	55.6	2.1	83.3
Mowat et al. 2011 [85]	Overall Guideline	77.8	72.2	59.4	91.7	37.5	50.0
	CAM Section	77.8	33.3	47.9	94.4	2.1	50.0

Overall scaled domain percentage scores varied across CPGs, ranging from 72.2 to 100.0% for scope and purpose, 30.6–91.7% for stakeholder involvement, 26.0–86.5% for rigour of development, 69.4–100% for clarity of presentation, 0.0–37.5% for applicability, and 0.0–100.0% for editorial independence. Average scaled domain percentages for overall CPGs, from highest to lowest, were clarity of presentation (90.3%), scope and purpose (91.5%), editorial independence (57.0%), rigour of development (54.7%), stakeholder involvement (56.7%), and applicability (14.6%).

Additionally, CAM scaled domain percentage scores varied across CPGs, ranging from 72.2 to 100.0% for scope and purpose, 8.3–94.4% for stakeholder involvement, 17.7–72.9% for rigour of development, 27.8–94.4% for clarity of presentation, 0.0–12.5% for applicability, and 0.0–100.0% for editorial independence. Average scaled domain percentages for CPGs’ CAM sections were, from highest to lowest, scope and purpose (91.5%), clarity of presentation (64.0%), editorial independence (57.0%), rigour of development (45.9%), stakeholder involvement (27.8%), and applicability (2.1%).

### Scope and purpose

Overall, all CPGs scored highly in scope and purpose, effectively communicating overall objectives and health questions in relation to the treatment and/or management of IBD. Different interventions, and their potential benefits and intended outcomes (e.g., induction of remission) were extensively described. The characteristics of the target population were also easily identifiable (e.g., “patients with mild-moderate UC” [47]).

### Stakeholder involvement

There was great variation in overall stakeholder involvement domain scores. All CPGs scored at least moderately well in describing overall guideline development group characteristics of members’ geographic locations, and institutional affiliations, with some CPGs scoring higher for further describing members’ disciplines (e.g., gastroenterologist, or methodologist) and/or specific roles in the group [37, 38, 43, 54, 56, 57, 59, 70, 71, 77, 83, 85]. Most CPGs clearly identified their target users, though some CPGs scored more poorly for not explicitly stating target users and not detailing how the CPG may be used [42, 59, 66, 70, 72, 73]. Some CPGs did not at all consider patients’ views and preferences in guideline development [47, 48, 59, 66, 70, 73], while others mentioned considering or emphasizing patient values but did not elaborate on what/how information was gathered [42, 43, 69, 71, 72, 83]. CPGs that scored moderately to very well additionally described methods and strategies used to capture patient values (e.g., literature review, patient advocate on guideline panel) [37, 38, 54, 56, 57, 68, 77, 85] and/or outcomes of gathered information (e.g., preference of avoiding medications’ adverse events over preventing disease recurrence) [53, 54, 56, 57, 68, 77].

In contrast, all CPGs but one [54] scored poorly for the CAM stakeholder involvement domain. CPGs’ CAM target user scores mirrored overall target user scores, with only some CPGs not explicitly stating target users and how to use the CPG [42, 59, 66, 70, 72, 73]. Other than one CPG [54], none of the CPGs involved CAM practitioners in their guideline development group nor described patient preferences regarding CAM therapies.

### Rigour of development

Most CPGs thoroughly described how systematic methods were used to find evidence (including CAM evidence) [37, 38, 43, 53, 56, 57, 59, 66, 68, 71, 77], though some CPGs did not include complete search strategies [59, 66]. Lower-scoring CPGs, with regards to systematic methods, either only described databases in which searches were performed [42, 47, 83, 85] or merely stated that literature searches were conducted [54, 70]. The lowest-scoring CPGs in systematic methods provided no evidence of a systematic literature search occurring [48, 69, 72, 73]. Surprisingly, not many CPGs explicitly stated all relevant inclusion/exclusion criteria (e.g., study designs, outcomes, population) for both overall and CAM evidence [37, 38, 53, 68, 77], though the other CPGs described the relevant population and at least partially described some studies that were included [42, 43, 47, 48, 54, 56, 57, 59, 66, 69–73, 83, 85]. Strengths and limitations of the body of evidence (including CAM evidence) were thoroughly described by seven CPGs [37, 38, 43, 53, 56, 57, 66, 68, 71]. The remaining eight CPGs reported on most aspects of strengths and limitations but were somewhat lacking details on study biases [42, 47, 48, 59, 69, 70, 72, 83] and/or the magnitude and consistency of results for benefits and harms [42, 48, 54, 73, 77, 83, 85]. Though lacking in CAM-specific considerations, most CPGs comprehensively described methods used for overall recommendation formulation [37, 38, 42, 43, 47, 56, 57, 59, 66, 71, 77, 83]. Accordingly, most CPGs incorporated a thorough consideration of health benefits versus harms in their overall and CAM recommendation formulation [37, 38, 42, 43, 47, 53, 54, 56, 57, 59, 66, 68, 71, 72, 77, 83, 85]. All CPGs also explicitly linked their recommendations (including CAM recommendations) with supporting evidence. Many CPGs merely described that external review occurred and/or described its purpose [37, 38, 43, 53, 54, 68, 70, 71, 77, 83]. No CPGs specifically detailed methods used or information gathered from an external review, nor did any CPG describe having CAM practitioners participate in an external review. Only four CPGs provided a procedure for updating overall CPGs (including CAM sections) with a specific timeframe [37, 38, 54, 77, 85].

### Clarity of presentation

Recommendations were specific and unambiguous for all of the overall CPGs and for most of the CAM sections, though a few CPGs were considerably vague regarding intent/purpose [47, 68, 69, 72, 73, 77]. All CPGs clearly presented different options for overall management of IBD. However, only five CPGs identified many CAM therapy options for IBD [48, 54, 59, 66, 85], whereas other CPGs only provided CAM recommendations against a therapy’s use [43, 56, 57, 68, 71, 77] or provided few CAM options [37, 38, 42, 47, 53, 69, 70, 72, 73, 83]. Key recommendations (both overall and CAM sections) were easily identifiable for all CPGs except one [73].

### Applicability

Applicability scaled domain percentages, for both overall CPGs and CAM sections, were generally poor. Only four CPGs described overall facilitators and barriers of recommendation implementation [66, 69, 77, 85], and no CAM facilitators or barriers were discussed in any CPG. Some CPGs provided limited advice or tools supporting recommendation implementation [37, 38, 54, 70, 77, 83, 85], mostly in links to compact guideline summaries or educational resources. Certain CPGs merely made mention of considering resource implications in formulating recommendations [37, 38, 42, 43, 72, 77, 83], while other CPGs’ recommendations additionally had some discussion of resource implications (e.g., recommendation caveats based on cost, interventions’ cost-effectiveness) [53, 56, 57, 59, 66, 68, 69, 71, 85]. No CPGs’ CAM sections discussed facilitators/barriers, provided advice/tools, or considered resource implications. Many CPGs had monitoring and/or auditing criteria for some recommendations [37, 38, 56, 57, 59, 66, 68, 77, 85], though in all instances they were lacking in detail. Few CPGs had monitoring and auditing criteria for CAM recommendations [54, 66, 70, 85].

### Editorial independence

For both overall CPGs and CAM sections, most CPGs identified funding sources, though only certain CPGs had explicit statements of no influence [43, 54, 56, 57, 66, 71, 83] while others lacked explicit statements [37, 38, 47, 48, 53, 59, 68, 77, 85]. Five CPGs entirely lacked funding body statements [42, 69, 70, 72, 73]. Similarly, regarding competing interests for both overall CPGs and CAM sections, most CPGs identified conflicts of interest, though only one CPG satisfactorily addressed these conflicts [54] while others did not [37, 38, 43, 47, 48, 56, 57, 60, 69, 71, 72, 77, 83, 85]. CPGs that declared no pertinent conflicts also scored well [53, 59, 66, 68], though the lowest-scoring CPGs lacked a competing interests section. [42, 73].

## Discussion

The objective of this review was to determine the quantity and assess the quality of CPGs providing CAM recommendations for the treatment and/or management of IBD. There were a wide range of CAM categories covered by different CPGs, though most CPGs had only a few CAM recommendations. The quality of 19 CPGs with CAM recommendations were assessed using the 23-item AGREE II instrument (where on each item’s Likert scale, 1 indicates strongly disagreeing that an item’s criteria were met and 7 indicates strongly agreeing that an item’s criteria were met). Domain scores differed greatly between different CPGs. Regarding overall guidelines, four CPGs [37, 38, 54, 56, 57, 77] scored ≥ 5.0 (and seven CPGs [42, 47, 48, 69, 70, 72, 73] scored ≤ 4.0) in both average appraisal score and average overall assessment. Regarding guidelines’ CAM sections, no CPGs scored ≥ 5.0 (and eleven [42, 47, 48, 59, 68–70, 72, 73, 83, 85] CPGs scored ≤ 4.0) in both average appraisal score and average overall assessment.

### Comparative literature

Although this review is, to our knowledge, the first to determine the quantity and assess the quality of CPGs providing CAM recommendations for the treatment and/or management of IBD, our findings can be compared with published reviews assessing both IBD CPGs as well as CAM recommendations in CPGs relating to other disease topics.

One study conducted a systematic review of IBD diagnosis and/or treatment CPGs, applying the AGREE II instrument and finding similar average scaled domain percentage findings: clarity of presentation (85.58%), scope and purpose (84.51%), editorial independence (62.02%), rigour of development (69.95%), stakeholder involvement (60.90%), and applicability (26.60%) [87]. The study’s authors concluded that the quality of most evaluated CPGs was acceptable, though there was room for improvement in the domains of stakeholder participation and applicability [87]. Another systematic review applied the AGREE II instrument to pharmacological therapy recommendations in IBD CPGs, though the pharmacological review differed considerably in domains of editorial independence (94.0%), applicability (45.8%), and stakeholder involvement (38.9%) [88] as compared to this present review. The pharmacological study discussed causes of heterogeneity between CPGs’ pharmacological recommendations (including varying efficacy of drugs in CD versus UC, special populations like pediatric patients, and potential developer bias in recommendation formulation), while suggesting that future guidelines could be improved through more refined recommendations based on target population characteristics (e.g., appropriate remission recommendations for severe UC adult patients may differ from moderate UC pediatric patients) [88]. A third study systematically reviewed diagnostic approaches in IBD CPGs and observed heterogeneity in diagnosis recommendations, while identifying domains of stakeholder involvement, rigour of development, and applicability as areas of improvement [89]. Finally, a systematic review examined the conflicts of interest and quality of evidence used for recommendations present in IBD CPGs, where authors noted considerable variation in recommendations’ evidence quality and numerous conflicts of interest in many CPGs [90].

This present review’s CAM section results can be compared to the findings of a systematic review that examined CPGs focused primarily on CAM therapies (e.g., herbal medicine, acupuncture, spinal manipulation) [91]. The CAM study had markedly different average scaled domain percentage findings: scope and purpose (83.3%), clarity of presentation (85.3%), editorial independence (60.1%), rigour of development (61.2%), stakeholder involvement (52.0%), and applicability (20.7%) [91]. Nonetheless, the CAM study similarly noted the paucity of high-quality CAM CPGs and variation in quality across domains [91]. Other studies assessing quality of CPGs’ CAM recommendations (using AGREE II) across various diseases/conditions (e.g., rheumatoid arthritis and osteoarthritis, colon cancer, multiple sclerosis, anxiety, depression) had similar trends in average scaled domain percentages of CAM sections, with clarity of presentation and scope and purpose domains tending to score higher, and stakeholder involvement and applicability tending to score lower [92–96].

Overall, this study revealed that there are few high-quality CPGs that comprehensively cover CAM therapy recommendations on IBD treatment and/or management. Of the 19 evaluated CPGs, 12 had only one or two CAM recommendations [37, 38, 42, 43, 47, 69–73, 77, 83, 85]. Many of these CPGs’ CAM recommendations were based on low-quality evidence [43, 68, 70, 71], or they were recommendations indicating knowledge gaps or neutral statements [47, 66]. Of seven CPGs with three or more CAM recommendations, four CPGs’ CAM recommendations consisted almost entirely of neutral/open recommendations [53, 54, 59, 69], generally due to knowledge gaps [53] or insufficient evidence [59, 69] for recommendations in favour of a given CAM therapy. For another CPG, two out of three CAM recommendations had very-low quality of evidence, and all three recommendations were against CAM therapy use [56, 57]. This study also found how the quality of CPGs’ CAM sections varied within each guideline (throughout different AGREE II domains) and between different guidelines.

There is a dearth of high-quality CAM research to support informed decision making on CAM use among healthcare professionals and patients. Challenges to CAM research include the absence of quality control and regulations on herbal supplements [10], challenges with blinding of physical interventions (e.g., acupuncture) or mind-body techniques (e.g., yoga) in study design [10], lack of funding [97], and bias against CAM research [97]. Despite these challenges, the use of CAM is highly prevalent among patients with IBD [10, 19–23]. Many patients with IBD also do not disclose their use of CAM to healthcare professionals, while many healthcare professionals have limited knowledge of CAM [10]. Altogether, patients’ hesitancy/inability to consult their healthcare provider on CAM therapies may negatively impact patient care and may be damaging to shared and informed decision making. A greater availability of high-quality CAM recommendations in CPGs, however, may present an opportunity for healthcare professionals to confidently provide informed advice on CAM use. Given the varying quality of CAM recommendations in CPGs, future development or updating of IBD CPGs can improve on guidelines’ CAM sections. One domain that could be improved on for future CPGs is stakeholder involvement, as most CPGs’ guideline development groups lacked CAM experts that may be knowledgeable of more therapies relevant to IBD treatment and/or management (who may help to increase the quantity of CAM recommendations). Similarly, incorporating patients’ views and preferences on CAM as part of the guideline development process can better inform healthcare professionals on shared care and decision-making principles [98]. For instance, the development of a CPG for the management of increased intestinal permeability [99] was informed by a cross-sectional survey of patient behaviours and preferences (which included questions about naturopathic practitioners and dietary supplements), which allowed the guideline to discuss the discrepancies between patients’ most commonly used dietary supplements and current evidence-based recommendations. Additionally, the same CPG had a diverse guideline development group that involved CAM experts, including naturopathic practitioners and integrative medicine practitioners, which allowed for the opportunity to consider the concordance between published evidence and clinical practice on managing increased intestinal permeability [99]. Ultimately, incorporating feedback from experts and patients may help increase uptake of the CPG among these target users [99, 100] Applicability is another domain that could be further improved upon due to the lack of tools in CPGs for clinicians to use to implement CAM recommendations into patient care plans and monitor therapy efficacy. One way to combat this would be for CPGs to include additional resources such as guides on facilitating CAM use discussions with patients, flow chart and algorithm versions of CPGs for deciding which CAM therapy is the most appropriate in a given situation, and patient versions of CPGs [101, 102]. Regarding rigour of development, given how many CPGs did not describe search strategies with many CAM terms (if described at all), developers may consider including more CAM terms in literature searches to potentially yield a greater body of CAM evidence for recommendation formulation. The AGREE II instrument can be used to identify criteria important for guideline reporting [31]. Furthermore, there exists other frameworks and checklists to help guide CPG development [103–105].

#### Strengths and limitations

One strength of this study is the use of systematic methods in identifying eligible CPGs for the treatment and/or management of IBD, though it is possible our search did not identify all relevant CPGs. Another strength is the use of the AGREE II instrument, which is widely accepted as the gold standard tool for evaluating CPGs [31–33]. A corresponding limitation, however, is how CPGs with CAM recommendations were evaluated by only two appraisers, rather than four appraisers as recommended by the AGREE II manual [31–33]. This limitation was partially addressed, however, by JYN, MCW, and HL conducting a pilot test to standardize scoring, where three different non-IBD CPGs were independently appraised before results were discussed to achieve consensus on how to apply the AGREE II instrument. Furthermore, independent appraisals of the 19 IBD CPGs with CAM recommendations by MCW and HL were followed by meetings and discussions with JYN to resolve uncertainties, while making sure to not change legitimate score discrepancies.

## Conclusions

The present study identified 49 CPGs published on IBD treatment and/or management since 2011, of which 19 CPGs made recommendations on CAM therapies such as probiotics, dietary and herbal supplements, fecal microbial transplantation, and mind-body medicine. Evaluation of these 19 CPGs with the AGREE II tool revealed variable quality within and across CPGs. Most CPGs had substantially lower CAM section AGREE II scores, as compared to non-CAM treatments in overall CPGs, where only one CPG was recommended for use by both appraisers. For future IBD guideline development and updates, CPGs with lower scaled domain percentages (for both overall and CAM-specific sections) could be improved with reference to the AGREE II instrument, as well as other guideline development resources. The general low quality of IBD CPGs’ CAM sections and low quantity of CAM recommendations in most CPGs presents a barrier to informed decision-making on CAM therapies among patients and healthcare professionals. Overall, future IBD guideline development would greatly benefit from improving CPGs’ CAM sections, specifically with regard to considering patients’ views on CAM, collaborating with CAM experts, providing CAM resources for patients and clinicians, and incorporating a greater quantity and quality of CAM evidence and recommendations.

**Fig. 1:**
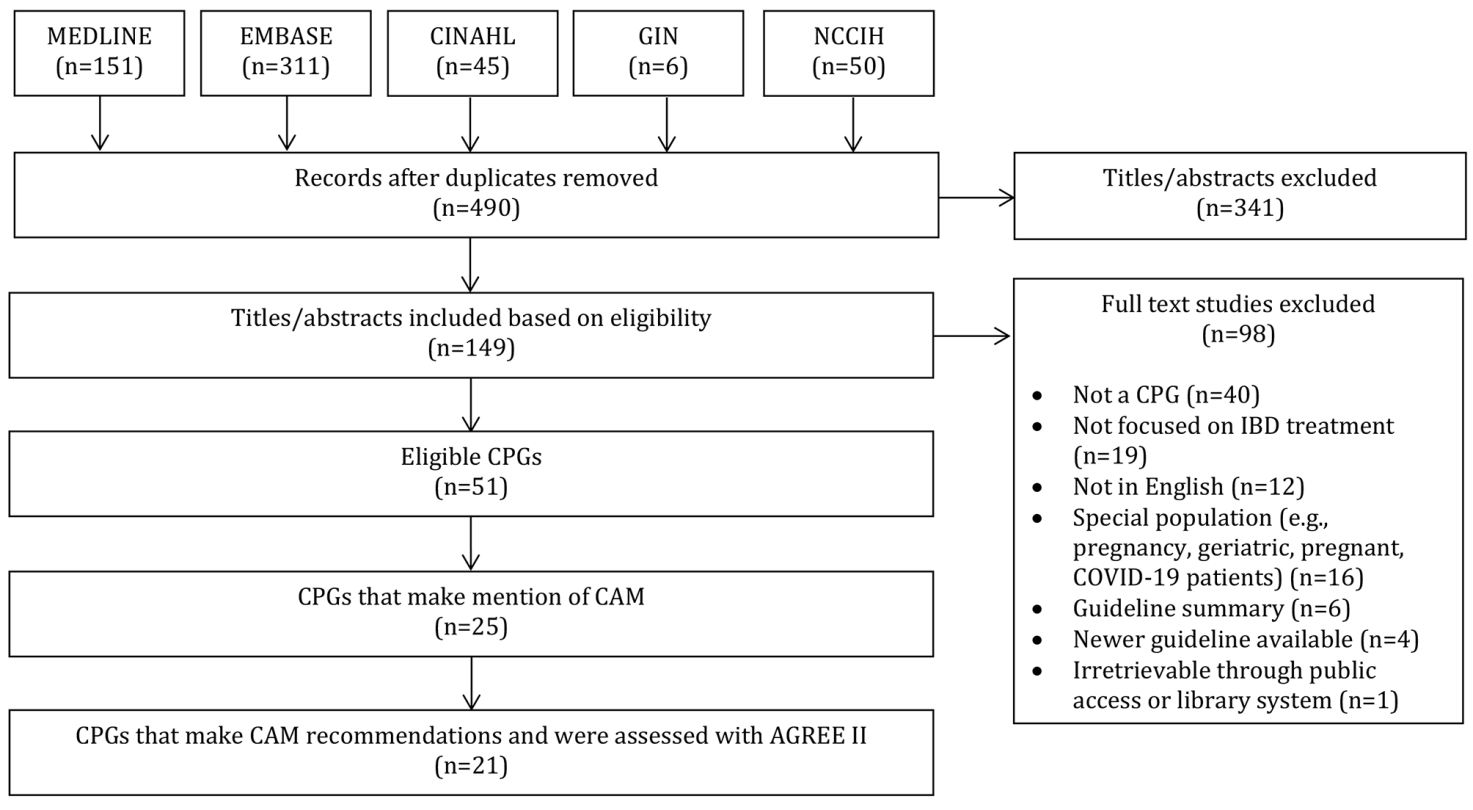
PRISMA Diagram

**Fig. 2:**
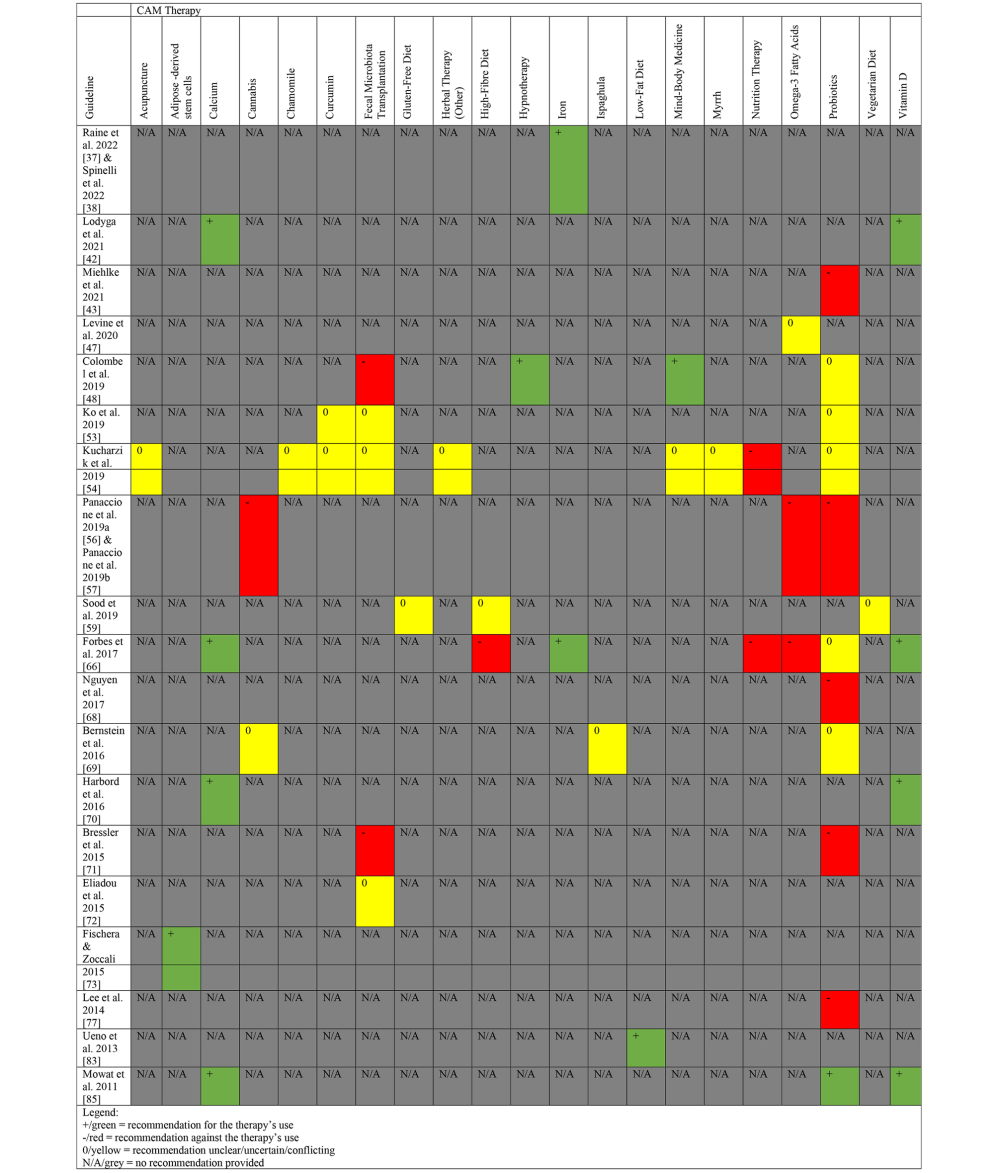
Summary of CAM Recommendations in Clinical Practice Guidelines

## Electronic supplementary material

Below is the link to the electronic supplementary material.

Supplementary Material 1

Supplementary Material 2

## Data Availability

All relevant data are included in this manuscript.

## Abbreviations

AGREE II: Appraisal of Guidelines for Research and Evaluation II
CAM: complementary and alternative medicine
IBD: Inflammatory bowel disease
UC: Ulcerative colitis
CD: Crohn’s disease
NCCIH: National Center for Complementary and Integrative Health
PICO: Patients, Intervention, Comparison and Outcomes
PRISMA: Preferred Reporting Items for Systematic Reviews and Meta-Analyses
