# Supplementary material for: Complementary and alternative medicine mention and recommendations in inflammatory bowel disease guidelines: systematic review and assessment using AGREE II
Source: BMC Complement Med Ther. 2023 Jul 11;23:230. doi: 10.1186/s12906-023-04062-0 (PMC10334672; doi:10.1186/s12906-023-04062-0)
Supplement: Supplementary file 1 — Supplementary Material 1 [file 12906_2023_4062_MOESM1_ESM.docx]

## Supplementary File 1: MEDLINE Search Strategy for Inflammatory Bowel Disease Clinical Practice Guidelines Executed May 19, 2022

| Database: Ovid MEDLINE(R) and Epub Ahead of Print, In-Process, In-Data-Review & Other Non-Indexed Citations, Daily and Versions <1946 to May 19, 2022>  Search Strategy:  --------------------------------------------------------------------------------  1 inflammatory bowel disease.mp. or Inflammatory Bowel Diseases/ (58391)  2 crohn's disease.mp. or Crohn Disease/ (59907)  3 ulcerative colitis.mp. or Colitis, Ulcerative/ (54601)  4 or/1-3 (123216)  5 limit 4 to ("all infant (birth to 23 months)" or "all child (0 to 18 years)" or "newborn infant (birth to 1 month)" or "infant (1 to 23 months)" or "preschool child (2 to 5 years)" or "child (6 to 12 years)" or "adolescent (13 to 18 years)") (22127)  6 4 not 5 (101089)  7 limit 6 to (english language and humans and yr="2011-current" and (guideline or practice guideline)) (151)  *************************** |
| --- |
